# Influenza, pneumococcal, and COVID-19 vaccination coverage and hesitancy in adults with psychiatric disorders

**DOI:** 10.3389/fpsyt.2025.1566348

**Published:** 2025-04-22

**Authors:** Vasfiye Demir Pervane, Betül Uyar, Pakize Gamze Erten Bucaktepe

**Affiliations:** ^1^ Family Medicine Department, Faculty of Medicine, Dicle University, Diyarbakir, Türkiye; ^2^ Psychiatry Department, Faculty of Medicine, Dicle University, Diyarbakir, Türkiye

**Keywords:** psychiatric disorder, vaccine, vaccine hesitancy, influenza, influenza vaccine, pneumococcus, pneumococcal vaccine, COVID 19

## Abstract

**Background/Objectives:**

Patients with psychiatric disorders have high mortality and morbidity rates from infectious diseases, but low vaccination rates compared to the normal population. This study aimed to evaluate the vaccination rates for influenza, pneumococcal, and COVID-19 vaccines, and the levels of vaccine hesitancy among individuals with psychiatric disorders.

**Methods:**

The study was a cross-sectional study among patients with psychiatric disorders. Participants’ vaccination statuses for influenza, pneumococcal, and COVID-19 vaccines during the pandemic were assessed, along with their vaccine hesitancy levels using a vaccine hesitancy scale. Data were collected between 01.03.2024 and 27.11.2024.

**Results:**

The study included 500 patients diagnosed with psychiatric disorders. Only 3.6% of the participants had received the influenza vaccine in the previous year, 3.0% reported regular influenza vaccinations, and 76.2% had received the COVID-19 vaccine during the pandemic. Among the participants at risk for pneumococcal infection (14%), the vaccination rate was only 2%. Patients with attention deficit hyperactivity disorder (ADHD) (45.0%) and anxiety disorder (17.2%) had the highest rates of regular influenza vaccination, while those with psychosis (13.6%) and depression (14.6%) had the lowest (p=0.010). COVID-19 vaccination rates during the pandemic were highest in ADHD (90.0%), bipolar disorder (81.1%), and depression (80.8%), and lowest in psychosis (54.5%) and obsessive-compulsive disorder (64.3%)(p=0.002). Women (p=0.001), participants with below university education levels (p=0.009), and patients with psychosis showed greater vaccine hesitancy. Patients with ADHD and bipolar disorder had the most positive attitudes toward vaccination (p=0.021). Positive attitudes were also linked to recent or regular influenza vaccinations and COVID-19 or pneumococcal vaccinations (p<0.05).

**Conclusions:**

Low vaccination rates and high vaccine hesitancy in psychiatric patients, particularly those with psychosis, necessitate targeted strategies to improve immunization coverage in this population.

## Introduction

Psychiatric disorders are important public health problems and are also common in society. In a global meta-analysis, it was reported that 29.2% of the global population experienced a mental disorder at least once in their lifetime, and 17.6% met the diagnostic criteria for a disease (1).

Due to their physical and mental health, individuals with psychiatric disorders are more susceptible to diseases (2–5). When all causes of death are taken into account, mortality rates were significantly higher in patients with psychiatric disorders compared to the control group, while the median years of potential life lost was 10 years (6). Inequalities in their use of and access to health services (7), their lower utilization of primary care services (8), and their lower utilization of preventive healthcare compared to patients without a psychiatric diagnosis (9) may render them more susceptible to diseases. While increased stress has been generally associated with a tendency to increased risk of infection and symptomatic illness (10), in addition to the presence of chronic stress in these individuals, genetic components in susceptibility to infection are mentioned, and it has been shown that the occurrence of infections in individuals with mental illness may be partially genetically directed (5).

Individuals with psychiatric disorders are at risk for infectious diseases (2–6, 11). It has been reported that the risk of dying from infections is 2.71 times higher and the risk of dying from respiratory tract infections is 3.27 times higher in people with serious mental illness (11). Furthermore, obesity, type 2 diabetes, cardiovascular diseases, and respiratory tract diseases, which are more prevalent in patients with severe psychiatric disorders compared to the general population, have the potential to exacerbate the outcomes of respiratory tract infections (7, 12). Seminog defined severe mental illness as a risk factor for pneumococcal diseases, lobar pneumonia, pneumococcal pneumonia, pneumococcal septicemia, and meningitis (2). In the Nilsson study, it was shown that influenza/pneumonia and sepsis-related mortality rates and hospitalizations were higher in patients with psychiatric diagnoses (3). In a cohort study conducted in the USA, it was reported that persons with serious mental illness were more likely to be hospitalized or to die after a coronavirus disease-2019 (COVID-19) diagnosis compared with persons without serious mental illness (13). In a cohort study evaluating COVID-19 infection and mortality in patients with psychiatric diagnoses, it was reported that schizophrenia patients had approximately 4 times, those with mood disorders 2.76 times, and those with anxiety disorders 2.39 times higher mortality rates than the reference group (4). In a systematic review and meta-analysis, it was mentioned that individuals with schizophrenia had a sevenfold significantly higher mortality rate from pneumonia than the general population (14) and in another, those with bipolar disorder had a 3.18 times higher mortality risk due to respiratory system infections (15).

Vaccines are one of the most effective tools in the fight against infectious diseases (16). Despite the evidence of the efficacy and safety of vaccines, vaccine hesitancy is recognized by the WHO as one of the 10 greatest global threats (17). Vaccine hesitancy in patients with mental illness can jeopardize vaccine uptake, which can pose a critical public health problem. Studies show that vaccination rates are lower in this patient group (18–24). In Lorenz’s study, influenza vaccination rates in patients with psychiatric diagnoses were reported to be lower than in the general population (19). Influenza vaccination uptake among target groups in Spain was below desirable levels and has not improved significantly (20).

During the COVID-19 pandemic, research suggested that individuals with psychiatric disorders had lower vaccination rates compared to the general population (21, 22, 24). In a retrospective cohort study evaluating data from 23.4 million adults in the UK, it was found that those with mental illness were alarmingly less vaccinated against COVID-19 compared to those with other chronic diseases (23). In a study evaluating COVID-19 vaccination rates in patients with severe mental illness during the pandemic in Turkey, it was reported that vaccination rates were lower than in the general population (24). During the pandemic period, it was shown that hospitalization rates increased in schizophrenia patients as the pandemic progressed and survival rates decreased (25). During the COVID-19 pandemic, it was recommended that people with severe mental illness be prioritized as a vulnerable group for COVID-19 vaccination due to increased mortality and morbidity rates due to COVID-19 and accompanying aggravating factors, and in some countries, this patient group was prioritized for vaccination (26–28). It is recommended that people with severe mental illness should be routinely vaccinated with pneumococcal and influenza vaccines and prioritized for vaccination (2, 3, 11).

Individuals with psychiatric diagnoses may experience vaccine hesitancy, which can become more complex due to additional complicating factors such as their perceptions of their current diagnosis. In a US study investigating associations between psychiatric morbidity and COVID-19 vaccine hesitancy, vaccine hesitancy was more prevalent across all psychiatric comorbidities (22). Patients with schizophrenia, in particular, tend to be less vaccinated (21, 25) and characteristics such as age and education can be risk factors for vaccination hesitation (29). In a study conducted in Turkey, it was determined that having schizophrenia increased the risk of non-vaccination 2.7 times (30). In a study conducted in Japan, it was shown that individuals with moderately severe or severe levels of depression were more ambivalent about COVID-19 vaccination (31). In the USA, it was found that adults with anxiety and depression were less vaccinated compared to those without anxiety and depression and had more concerns about the efficacy of vaccines, side effects, and trust in the government (32).

In this study, we aimed to reveal influenza, pneumococcal, and COVID-19 vaccination rates, vaccine hesitancy, and related factors in adult individuals with psychiatric disorders. Revealing the vaccination rates, characteristics of vaccine hesitancy, and related factors in this patient group will contribute to the development of effective interventions at both individual and social levels and allow for the development of coping strategies against infectious diseases for individuals with psychiatric disorders, who can be considered an at-risk group for infectious diseases.

## Materials and methods

This study was designed as a cross-sectional analytical study, and the Strengthening the Reporting of Observational Studies in Epidemiology (STROBE) guidelines were utilized in the preparation of the study (33). Ethics committee approval was obtained for the study (date: 14.02.2024 no: 114) The study was conducted in accordance with the Declaration of Helsinki.

Within the scope of the study, patients who were diagnosed with psychiatric disorders by a psychiatrist who attended the Dicle University Faculty of Medicine Hospital Mental Health Diseases and Family Medicine outpatient clinics, which are the two outpatient clinics most frequently visited by patients with psychiatric diagnosis, were invited to participate in the study. The number of people to be included in the study was calculated with the sample size calculator. By taking a medium effect size (0.5) p ≤ 0.05 and the pairwise hypothesis of 0.95 (95%) power at a 95% confidence interval into account, the minimum sample size was determined to be 373 people. Data were collected between 01.03.2024 and 27.11.2024. The minimum number of participants for the study was 373, but the study was terminated when the number of participants was reached to allow the analysis to be carried out among the sub-diagnoses, and therefore the total number of participants was 500. In addition, psychiatric patients repeatedly attended the outpatient clinic due to their treatment, and the study was terminated before the planned time to prevent repeated study participation of the same participants. 

A face-to-face questionnaire was used for data collection, and written informed consent was obtained from the participants. The inclusion criteria included volunteering to participate in the study, being over 18 years of age, and having a psychiatric diagnosis. Patients diagnosed with schizophrenia and other psychotic disorders, bipolar disorder and related disorders, depression, anxiety disorders, obsessive-compulsive personality disorders (OCD), and attention deficit hyperactivity disorder (ADHD) were included in the study.

### Variables

Participants’ sociodemographic characteristics (age, sex, educational status, marital status, and number of children), psychiatric diagnoses, chronic diseases, care status, and adult vaccination characteristics (influenza, COVID-19, and pneumococcal) were questioned and recorded. The Vaccine Hesitancy Scale was used as a scale. The Vaccine Hesitancy Scale is a valid and reliable scale consisting of nine items, the validity and reliability of which were established by Luyten et al. (34) and adapted into Turkish by Yılmaz et al. (35). The scale features a 5-point Likert-type response system, ranging from strongly disagree to strongly agree. The total score on the scale ranges from 9–45 points. The higher the score obtained from the scale, the lower the vaccine hesitation.

### Statistical analysis

The Statistical Package for the Social Sciences (SPSS), version 27.0 for Windows (SPSS Inc., Chicago, USA), a computer package program, was used for statistical analysis of the research data. As descriptive statistics, categorical variables were presented as numbers and percentages, and continuous variables were presented as mean ± standard deviation (minimum–maximum value) and median (IQR) according to distribution characteristics. Histogram and skewness-kurtosis were used to determine whether the data fit the normal distribution. In the comparison of two independent groups, the independent groups t-test or Mann-Whitney U test was used, and one-way analysis of variance (ANOVA) was used for more than two groups. The homogeneity of the variance in multiple groups was checked with Levene’s test, and since the variance was found to be homogeneous, Tukey’s *post hoc* test was used to determine which group was the source of the significance. The relationship between continuous data was evaluated by Pearson correlation analysis. Chi-square analysis was used to determine whether there was a statistical difference between two qualitative variables. Bonferroni-corrected *post hoc* analysis was used to determine which category was the source of significance in categorical data with more than two groups between which significance was found. The reliability (Cronbach α) coefficient of the scale for this sample was also calculated. The Cronbach α value of the scale was found to be 0.871 in our study. Hypotheses were two-way, and p<0.05 was considered statistically significant.

## Results

A total of 500 patients with psychiatric diagnoses participated in the study, and 62.2% of the participants were female, 50.0% were married, 50.0% had children, and 61.2% had a high school education level or higher (**Table 1**). Most of the participants were diagnosed with anxiety disorder, with 45.4% (n=227), and depression with 26.0% (n=130). These diagnoses were followed by psychosis (8.8%), OCD (8.4%), bipolar disorder (7.4%), and ADHD (4%). The mean age of all participants was 35.5 years, with the lowest mean age in patients with ADHD (25.1) and the highest mean age in patients with anxiety disorder (36.2). Furthermore, 96% of the participants stated that they were able to perform their own self-care. Of the participants, 132 (26.4%) stated that they had a chronic disease, and 127 (25.4%) stated the type of chronic disease. Among those with chronic diseases, 49% (n=79) had endocrine diseases, 24.8% (n=40) had cardiovascular diseases, 11.1% (n=18) had thoracic diseases, 6.8% (n=11) had neurological diseases, 5.5% (n=9) had gastroenterology, and 2.4% (n=4) had rheumatologic diseases.

**Table 1:** Descriptive characteristics of the participants.

Descriptive characteristics		n	%
Sex	Female	311	62.2
	Male	189	37.8
Education status	Illiterate	37	7.4
	Literate	27	5.4
	Primary school	81	16.2
	Middle school	49	9.8
	High school	138	27.6
	University and higher	168	33.6
Marital status	Married	250	50.0
	Not married	250	50.0
Child presence	No	250	50.0
	Yes	250	50.0
Diagnosis	OCD	42	8.4
	Anxiety disorder	227	45.4
	Depression	130	26.0
	Psychosis	44	8.8
	Bipolar disorder	37	7.4
	ADHD	20	4.0
To be able to do their own self-care	No	4	0.8
	Partially	16	3.2
	Yes	480	96.0
Chronic disease	No	368	73.6
	Yes	132	26.4
Chronic disease classification*	Chest diseases	18	11.9
	Endocrine	79	49.0
	Neurology	11	6.8
	Cardiovascular system	40	24.8
	Rheumatology	4	2.4
	Gastroenterology	9	5.5
	Total	161	100
		Mean ± SD (min-max)	
Number of children	Total	3.1 ± 1.6 (1–9)	
Age	OCD	33.9 ± 9.6 (18–55)	
	Anxiety disorder	36.2 ± 13.1 (18–83)	
	Depression	37.2 ± 13.6 (18–73)	
	Psychosis	33.3 ± 9.6 (18–54)	
	Bipolar disorder	35.5 ± 34.6 (18–66)	
	ADHD	25.1 ± 4.5 (18–33)	
	Total	35.5 ± 12.7 (18–83)	

OCD, obsessive-compulsive disorder; ADHD, attention deficit hyperactivity disorder; *More than one answer was given.

When we examined the vaccination status of all participants, only 3.6% (n=18) stated that they had received the influenza vaccine during the study year, while 3.0% (n=15) stated that they had received a regular influenza vaccine every year (**Table 2**). While 76.2% (n=381) had received the COVID-19 vaccine during the pandemic, the most common vaccine was the BioNTech vaccine, with 68.7% (n=262), and only 1.9% (n=9) had received the COVID-19 vaccine during the study year. While 14% (n=70) of all participants were in the at-risk group for the pneumococcal vaccine, the rate of pneumococcal vaccination was only 2%.

**Table 2:** Distribution of vaccination status of participants according to diagnoses.

		Anxiety disorder		Depression		Psychosis		OCD		Bipolar disorder		ADHD		Total	
		n	%	n	%	n	%	n	%	n	%	n	%	n	%
Getting vaccinated against influenza during the study year	Yes	11	4.8	2	1.5	2	4.5	0	0	1	2.7	2	10	18	3.6
	No	216	95.2	128	98.5	42	95.5	42	100	36	97.3	18	90	482	96.4
Regular influenza vaccination every year	Yes	9	4.0	2	1.5	2	4.5	0	0	1	2.7	1	5.0	15	3.0
	Sometimes	30	13.2	17	13.1	4	9.1	3	7.1	5	13.5	8	40.0	67	13.4
	No	188	82.8	111	85.4	38	86.4	39	92.9	31	83.8	11	55.0	418	96.4
Getting vaccinated against COVID-19 during the study year	Yes	0	0	1	0.8	4	9.1	0	0	3	8.1	1	5	9	1.9
	No	227	100	129	99.2	40	90.9	42	100	34	91.9	19	95	491	98.2
COVID-19 vaccination during the pandemic	Yes	177	78.0	105	80.8	24	54.5	27	64.3	30	81.1	18	90	381	76.2
	No	50	22.0	25	19.2	20	45.5	15	35.7	7	18.9	2	10	119	23.8
COVID-19 vaccine strain during the pandemic	Sinovac	25	14.2	15	14.3	3	12	3	11.1	4	13.4	3	38.9	53	13.4
	BioNTech	124	70.0	70	66.7	17	68	22	81.4	21	70	8	44.4	262	68.7
	Sinovac+ BioNTech	29	15.8	20	19.0	5	20	2	7.4	5	16.6	7	16.7	68	17.8
Pneumococcal vaccination status	Yes	9	4.0	0	0	1	2.3	0	0	0	0	0	0	10	2.0
	No	218	96.0	130	100	43	97.7	42	100	37	100	20	100	490	98.0
Being in the at-risk group for pneumococcal vaccination	Yes	38	16.7	21	16.2	3	6.8	3	7.1	5	13.5	0	0	70	14.0
	No	189	83.3	109	83.8	41	93.2	39	92.9	32	86.5	20	100	430	86.0
Pneumococcal vaccine type	CPV-13	2	22.2	0	0	1	100	0	0	0	0	0	0	3	30
	PPA-23	6	66.6	0	0	0	0	0	0	0	0	0	0	6	60
	Unknown or not remembered	1	11.1	0	0	0	0	0	0	0	0	0	0	1	10

ADHD, attention deficit hyperactivity disorder; OCD, obsessive-compulsive disorder; COVID-19, coronavirus disease-2019; CPV-13, conjugated pneumococcal vaccine-13; PPA-23, polysaccharide pneumococcal vaccine-23.

When the vaccination status was evaluated according to the diagnoses, it was found that 10% of those diagnosed with ADHD, 4.8% of those diagnosed with anxiety disorder (n=11), and 4.5% of those with psychosis (n=2) had received the influenza vaccine this year; these rates were 1.5% (n=2) for depression and 2.7% (n=1) for bipolar disorder, and those diagnosed with OCD did not receive any influenza vaccine during the study year. While 55% of those diagnosed with ADHD stated that they had received a regular influenza vaccination every year, 92.9% of those diagnosed with OCD and 86.4% of those with psychosis stated that they did not receive a regular influenza vaccination. During the pandemic, 90% (n=18) of those diagnosed with ADHD, 80.8% (n=105) of those diagnosed with depression, and 81.1% (n=30) of those diagnosed with bipolar disorder stated that they had received the COVID-19 vaccine. Patients diagnosed with psychosis (54.5% (n=24)) and OCD (64.3% (n=27)) were the least likely to have received the COVID-19 vaccine during the pandemic. While the rate of being in the risk group for pneumococcal vaccination was between 7.1% and 16.7% among the diagnostic groups (except for those with ADHD with 0%), the pneumococcal vaccination rates were 4.0% (n=9) for anxiety disorder and 2.3% (n=1) for patients diagnosed with psychosis, and no patients in the other diagnostic groups were vaccinated with the pneumococcal vaccine. The most commonly administered pneumococcal vaccine was the polysaccharide pneumococcal vaccine 23 (PPA-23), with 60% (n=6) of all patients and 66.6% (n=6) of patients with anxiety disorder who were in the at-risk group for the pneumococcal vaccine receiving the PPA-23 pneumococcal vaccine.

The descriptive characteristics of the participants’ regular influenza vaccination status and COVID-19 vaccination status during the pandemic were compared and are shown in **Table 3**. It was found that there was a statistically significant difference (p=0.010) in the comparison of psychiatric diagnoses and those who received the influenza vaccine every year or sometimes compared to those who did not; the psychiatric diagnosis group that received the influenza vaccine the most was the patient group with ADHD (45.0%) and then the patient group with anxiety (17.2%), while the patients diagnosed with psychosis (13.6%) and depression (14.6%) received the least. The Bonferroni correction showed that the difference was due to the difference between participants with ADHD and other diagnostic groups. Since the number of people who received influenza vaccination and COVID-19 vaccination this year fell to numbers that could not be statistically applied in subgroups, their evaluations were not included in the table.

**Table 3:** Comparison of the descriptive characteristics of the participants and their status of receiving the influenza vaccine every year and the COVID-19 vaccine during the pandemic.

		Regular influenza vaccination			COVID-19 vaccination during the pandemic		
		No	Yes-Sometimes		No	Yes	
		n %*	n %*	p**	n %*	n %*	p**
Diagnosis	OCD	39^a^ (92.9)	3^a^ (7.1)	0.010	15^a^ (35.7)	27^a^ (64.3)	0.002
	Anxiety disorder	188^a^ (82.8)	39^a^ (17.2)		50^a^ (22.0)	177^a^ (78.0)	
	Depression	111^a^ (85.4)	19^a^ (14.6)		25^a^ (19.2)	105^a^ (80.8)	
	Psychosis	38^a^ (86.4)	6^a^ (13.6)		20^a^ (45.5)	24^b^ (54.5)	
	Bipolar disorder	31^a^ (83.8)	6^a^ (16.2)		7^a^ (18.9)	30^a^ (81.1)	
	ADHD	11^a^ (55.0)	9^b^ (45.0)		2^a^ (10.0)	18^a^ (90.0)	
Chronic disease	No	314 (85.3)	54 (14.7)	0.082	93 (25.3)	275 (74.7)	0.197
	Yes	104 (78.8)	28 (21.2)		26 (19.7)	106 (80.3)	
Status of being in the at-risk group for pneumococcal vaccination	No	371 (86.3)	59 (13.7)	<0.001	106 (24.7	324 (75.3	0.268
	Yes	47 (67.1)	23 (32.9)		13 (18.6	57 (81.4	
Age	40 years and younger	296 (87.6)	42 (12.4)	<0.001	97 (28.7)	241 (71.3)	<0.001
	41 years and older	122 (75.3)	40 (24.7)		22 (13.6)	140 (86.4)	
Sex	Female	262 (84.2)	49 (15.8)	0.620	77 (24.8)	234 (75.2)	0.518
	Male	156 (82.5)	33 (17.5)		42 (22.2)	147 (77.8)	
Marital status	Married	205 (82.0)	45 (18.0)	0.334	60 (24.0)	190 (76.0)	0.916
	Single	213 (85.2)	37 814.8)		59 (23.6	191 (76.4)	
Education status	Primary school and lower	119 (82.1)	26 (17.9)	0.083	42 (29.0)	103 (71.0)	<0.001
	Middle school–high school	165 (88.2)	22 (11.8)		59 (31.6)	128 (68.4)	
	University and higher	134 (79.8)	34 (20.2)		18 (10.7)	150 (89.3)	
Child presence	No	212 (84.8)	38 (15.2)	0.469	61 (24.4)	189 (75.6)	0.753
	Yes	206 (82.4)	44 (16.4)		58 (23.2)	192 (76.8)	

*Row percentages, **Chi-Squared test.

OCD, obsessive-compulsive disorder; ADHD, attention deficit hyperactivity disorder; COVID-19, coronavirus disease-2019.

Different letters (superscript) indicate that the ratio differences between variables were statistically significant in the *post hoc* tests (Bonferroni corrected).

The descriptive characteristics of the participants were compared with the status of influenza vaccination every year and COVID-19 vaccination in the pandemic, and a statistically significant result was found between the diagnostic groups and the status of COVID-19 vaccination in the COVID-19 pandemic (p=0.002); the psychiatry diagnostic group that stated that they received the vaccine the most was ADHD (90.0%) and bipolar disorder (81.1%), followed by patients diagnosed with depression (80.8%). The group that received the least COVID-19 vaccinations was the group of patients with psychosis (54.5%), followed by the group of patients with OCD (64.3%), and the Bonferroni correction showed that the statistically significant difference was due to the difference between participants diagnosed with psychosis and other diagnostic groups. The rate of regular influenza vaccination among those in the at-risk group for the pneumococcal vaccine was lower (32.9%) than among those who were not (67.1%); however, the rate of regular influenza vaccination was higher among those in the at-risk group for the pneumococcal vaccine than among those not in the at-risk group (32.9% versus 13.7%), and this difference was statistically significant (p=<0.001). Among all participants, regular influenza (p<0.001) and pandemic COVID-19 vaccination rates were significantly higher in people over 40 years of age and those with a university education or higher (p<0.001).

The responses of all participants to the vaccine hesitancy scale questions are presented in **Table 4**. In total, 91.2% (moderately agree to strongly agree) of the participants stated that vaccines are important for their health, 91.0% (moderately agree to strongly agree) stated that vaccines are effective, and 93.6% (moderately agree to strongly agree) stated that vaccination is important for the health of others in the community. Furthermore, 74.8% (moderately agree to strongly agree) of the participants agreed with the statement that all vaccines in the vaccination program offered by the government to the community are beneficial and that new vaccines carry more risk than old vaccines. It was found that 80.8% (moderately agree to strongly agree) of all the participants found the information they received about vaccines from the government’s vaccination program credible and reliable, while 75.6% were concerned about the serious side effects of vaccines. It was found that 92.8% of the participants agreed with the statement that getting vaccinated is a good way to protect themselves from disease, and 93% of the participants agreed with the statement that they usually follow the recommendations of their doctor and health institution about vaccines.

**Table 4:** Responses of the participants to each scale item.

Scale Items	Strongly disagree n (%)	Disagree n (%)	Moderately agree n (%)	Agree n (%)	Strongly agree n (%)
Vaccinations are important for my health	11 (2.2)	33 (6.6)	92 (18.4)	213 (42.6)	151 (30.2)
Vaccines are effective	11 (2.2)	34 (6.8)	82 (16.4)	232 (46.4)	141 (28.2)
Being vaccinated is important for the health of others in my community	8 (1.6)	24 (4.8)	67 (13.4)	235 (47.0)	166 (33.2)
All vaccines offered by the government program in my community are beneficial	18 (3.6)	108 (21.6)	132 (26.4)	139 (27.8)	103 (20.6)
New vaccines carry more risks than older vaccines	19 (3.8)	107 (21.4)	138 (27.6)	175 (35.0)	61 (12.2)
The information I receive about vaccines from the vaccine program is reliable and trustworthy	20 (4.0)	76 (15.2)	152 (30.4)	157 (31.4)	95 (19.0)
Getting vaccines is a good way to protect myself from disease	8 (1.6)	28 (5.6)	84 (16.8)	239 (47.8)	141 (28.2)
I generally do what my doctor or healthcare provider recommends about vaccines	9 (1.8)	26 (5.2)	81 (16.2)	239 (47.8)	145 (29.0)
I am concerned about serious adverse effects of vaccines	32 (6.4)	90 (18.0)	131 (26.2)	157 (31.4)	90 (18.0)

The mean total scale score of all participants was 32.0 ± 6.4 (9–45). Participants’ scores on the scale and descriptive characteristics are compared and shown in **Table 5**. Considering that a decrease in the score obtained on the vaccine hesitancy scale reflects an increase in hesitancy towards vaccination, it was found that the scale scores of women (p=0.001) and those with less than a university education (p=0.009) were statistically significantly lower; that is, their attitudes were more negative. When psychiatric diagnosis and scale scores were compared (p=0.021), it was observed that there was a statistically significant difference; the lowest scale scores were in the group of patients with psychosis (30.4 ± 5.7), followed by patients with depression (31.1 ± 31.1) and anxiety disorder (32.2 ± 6.6). The highest scale scores were found in patients diagnosed with ADHD (35.5 ± 6.8) and bipolar disorder (33.0 ± 5.9).

**Table 5:** Comparative analysis of descriptive characteristics and vaccine hesitancy scale scores.

Characteristics		Scale score mean ± SD/Median (IQR)	t/z/f	p
Sex*	Female	31.3 ± 6.6	-3.271	0.001
	Male	33.2 ± 5.9		
Education Status**	Primary school and lower^a^	31.5 ± 6.7	4.781	0.009
	Middle school–high school^a^	31.3 ± 6.3		
	University and higher^b^	33.2 ± 6.1		
Marital status	Married	31.7 ± 6.7	-1.036	0.301
	Not married	32.3 ± 6.0		
Child presence*	Yok	32.5 ± 6.1	1.810	0.071
	Var	31.5 ± 6.7		
Diagnosis**	OCD^a,b^	32.8 ± 6.4	2.676	0.021
	Anxiety disorder^a,b^	32.2 ± 6.6		
	Depression^a^	31.1 ± 6.2		
	Psychosis^a^	30.4 ± 5.7		
	Bipolar disorder^a,b^	33.0 ± 5.9		
	ADHD^b^	35.5 ± 6.8		
Chronic disease*	No	32.3 ± 6.2	-1.325	0.187
	Yes	31.3 ± 6.9		
Able to do their own self-care***	No or partially	30.5 (11)	-1.312	0.189
	Yes	32.0 (8)		
In the at-risk group for pneumococcal vaccination*	No	32.1 ± 6.3	-0.636	0.525
	Yes	31.6 ± 7.1		
Getting vaccinated against influenza during the study year***	No	32.0 (8)	-2.665	0.008
	Yes	35.0 (6)		
Regular influenza vaccination every year*	No	31.7 ± 6.5	-2.495	0.013
	Yes	33.6 ± 5.8		
COVID-19 vaccination during the pandemic*	No	30.0 ± 6.6	-4.036	<0.001
	Yes	32.6 ± 6.2		
Getting vaccinated against COVID-19 during the study year***	No	32.0 (8)	-0.385	0.700
	Yes	34.0 (3)		
Pneumococcal vaccine status***	No	32.0 (8)	-2.526	0.012
	Yes	37.0 (9)		

*Independent Samples t Test, **One Way ANOVA, ***Mann-Whitney U Test.

OCD, Obsessive Compulsive Disorder; ADHD, Attention Deficit Hyperactivity Disorder.

COVID-19, Coronavirus Disease-2019; SD, Standard Deviation; IQR, Interquartile Range.

Different letters (superscript) indicate that the score differences between variables are statistically significant in *post hoc* tests (Türkiye).

When the vaccination status of the participants was compared with their scores on the vaccine hesitancy scale (**Table 5**), it was found that the scale scores of those who received the influenza vaccine during the study year (p=0.008), those who stated that they received the regular influenza vaccine every year (p=0.013), those who received the COVID-19 vaccine during the pandemic (p=<0.001), and those who received the pneumococcal vaccine (p=0.012) were statistically significantly higher.

No significant correlation was found between the scale score and age (r=-0.068, p=0.128) and number of children (r=-0.017, p=0.792). In addition, those who received Sinovac and BioNTech vaccines during the pandemic were compared, and the scale score of those who received only the Sinovac vaccine was 30.79, while the scale score of those who received only the BioNTech vaccine was 33.0, and the difference was statistically significantly higher (p=0.018).

## Discussion

Vaccination is one of the most important steps in the fight against infectious diseases and is of critical importance, especially for psychiatric patients who are at higher risk of infection, mortality, and morbidity. Although individuals with psychiatric disorders may be considered part of the at-risk group to be prioritized for vaccination, influenza and pneumococcal vaccination rates among these patients were found to be alarmingly low in our study. Regular influenza vaccination rates were significantly lower among patients with psychosis and depression compared to patients with other diagnoses, and COVID-19 vaccination rates were significantly lower among patients with psychosis and OCD during the pandemic. Women, participants with lower levels of education, and patients with psychosis showed more vaccine hesitancy. Positive attitudes were also associated with having recently or regularly been vaccinated against influenza and being vaccinated against COVID-19 or pneumococcal vaccine. Low vaccination rates and high vaccine hesitancy in psychiatric patients, especially those with psychosis, suggest the need for targeted strategies to improve vaccination coverage in this high-risk population.

Influenza has a negative effect on mortality and hospitalization rates in patients with psychiatric diagnoses (3); therefore, influenza vaccination is recommended in this patient group (11). Although influenza is a vaccine recommended to be administered annually, in this study, a very low rate of 3.6% of all participants stated that they received the influenza vaccine within a year, and 3.0% stated that they received the regular influenza vaccine. Low influenza vaccine coverage has also been reported in high-risk patient groups other than psychiatric disorders (20). Influenza vaccination rates have been found to be low among patients with psychiatric diagnoses in the literature; however, influenza vaccination rates were much higher in our study (19, 20, 36). In Greece, the influenza vaccination rates of patients with mental disorders between 2020 and 2022 were reported to be 43.2%, 39.8%, 40.7%, and 33.7%, respectively; the rate of those who received influenza vaccinations repeatedly over 3 years was 33.7% (36). A study conducted in the USA reported influenza vaccination rates of 28.4% and 24.2% in 2010 and 2011, respectively, lower than in the general population at that time (19). An ecological study analyzing data from 34 countries worldwide revealed an inverse correlation between influenza vaccine coverage and COVID-19 mortality (37) and recommended influenza vaccination to be administered primarily in at-risk groups during the pandemic (38). During and after the COVID-19 pandemic, there was an increase in influenza vaccination rates in adults in the general population compared to before (39). While the study in the USA was conducted before the pandemic (19), the study in Greece was conducted after the pandemic (36). Higher influenza vaccination rates after the pandemic may be associated with the impact of the pandemic; however, although our study was conducted 1 year after the official end of the pandemic (40), the fact that influenza vaccination rates were less than one-tenth of the rates reported in the Greek study is a remarkable result that is difficult to explain.

When the influenza vaccination status within 1 year was evaluated according to the diagnoses, it was found that the vaccination rates were very low in patients diagnosed with depression (1.5%), bipolar disorder (2.7%), psychosis (4.5%), and anxiety disorder (4.8%); no patient with OCD had received the influenza vaccine, and even in patients diagnosed with ADHD, the highest vaccination rate was 10%. Regular influenza vaccination rates were also significantly lower among patients with psychosis and depression compared to patients with other diagnoses. In the study conducted in Greece, higher rates were found among people with anxiety disorders (41.5%) and a depressive episode (41.1%), while the lowest rates were reported among patients with schizophrenia (26.8%), bipolar disorder (24.5%), and obsessive-compulsive disorder (22.2%) (36). Vaccination rates of patients with psychiatric diagnoses vary in the literature (41). Although respiratory tract infections have been associated with increased mortality and morbidity in patients with psychiatric diagnoses (2, 3, 11, 13), vaccination rates are generally reported to be lower in this patient group (18–24). Increased mortality rates reported, especially in patients with schizophrenia, emphasize the importance of vaccination in these patient groups (4, 13, 14). In the Lawrence study, it was reported that any mental health diagnosis increased the likelihood of influenza vaccination in the general sample, and the relationship between mental health diagnosis and the likelihood of influenza vaccination was stronger, especially in people with comorbidities (42). In Lawrence’s study, the fact that the patient group consisted of individuals over 65 years of age may be an important reason for increased influenza vaccine uptake. This age group is considered to be at risk for influenza due to their age and potential chronic diseases (43). Sociodemographic characteristics may have an impact on vaccine acceptance. Age, female sex, and some concomitant chronic diseases may increase influenza vaccine uptake. In a study conducted in Spain, while the rate of patients in the risk group defined for influenza was 19.2% above the age of 15 years, this rate increased to 54.4% above the age of 65 years and 41.6% with concomitant medical conditions (20). In this study, it was found that among all participants, the rate of regular influenza and pandemic COVID-19 vaccinations was significantly higher in people over the age of 40 and those with a university or higher education level. In a study conducted in Greece, being over 50 years of age, polypharmacy, and non-hospitalization to a psychiatry ward were positively associated with influenza vaccination (36). In addition, being 50 years of age or older and of the female sex were reported as predictor factors for CPV-13 vaccination. In Lorenz’s study, it was reported that people with education levels above high school tended to have fewer flu vaccinations because they thought that they would get the flu from more vaccines (19).

Patients with severe mental illness may be more susceptible to infectious diseases due to unhealthy lifestyle choices and medications that may accompany the associated metabolic comorbidities. Pneumococcal vaccination is recommended for individuals who are more vulnerable to pneumococcal infections, but having a psychiatric diagnosis is not included in the at-risk group (44). In a cohort study conducted by Seminog among patients with schizophrenia, bipolar disorder, depression, or anxiety, severe mental illness was considered a risk factor for pneumococcal disease, and an approximately 2.3-fold increased risk of pneumococcal disease was reported (2). It is recommended that these patient groups should be defined as a priority risk group for pneumococcal and influenza vaccination (2, 3, 11). In this study, while 14% of all participants were in the defined at-risk group for pneumococcal vaccination, the rate of pneumococcal vaccination was very low at only 2%. The most common type of pneumococcal vaccine reported to be administered was PPA-23 (60%), while the group that received the most pneumococcal vaccine was found to be patients with anxiety disorders. In a study conducted in Greece, the pneumococcal vaccination rates of patients with mental disorders were reported to be 28.8% for CPV-13 and 7.7% for PPA-23, and the highest vaccination rate was reported in patients diagnosed with depressive disorder (49.4%) (36). In our study, for pneumococcal vaccination, the rate of regular influenza vaccination was found to be lower in those in the at-risk group (32.9%) compared to those not in the at-risk group (67.1%); however, a statistically significant higher rate of regular influenza vaccination was found in those in the at-risk group for PPA-23 compared to those not in the risk group (32.9% versus 13.7%). The study in Greece reported that a person vaccinated with CPV-13 was 14 times more likely to have been vaccinated regularly against influenza within 1 year and 17.3 times more likely to have been vaccinated regularly against influenza within the last 3 years (36). In our study, the vaccination experiences of the participants were recorded by self-report, and health system records were not reviewed. The participants’ existing psychiatric diseases may have limited their recall of both vaccination status and vaccine type.

The COVID-19 pandemic caused high mortality and morbidity in patients with psychiatric disorders (4, 11, 13, 25); these patients had lower COVID-19 vaccine uptake (21–24), and due to increased poor clinical outcomes in patients with psychiatric disorders, it was recommended that these patients be prioritized during pandemic vaccinations (26–28). In our study, 76.2% of all participants reported having received a COVID-19 vaccine during the pandemic. While questioning the patients about their COVID-19 vaccination status, we did not ask about the number of vaccine doses, so the 76.2% rate corresponds to the number of people who had been vaccinated with at least one dose of any COVID-19 vaccine, and this rate was below the rate of at least one dose of vaccination (93.3%) during the pandemic period in Turkey (45). Maazerel reported that 93.0% of patients with psychiatric disorders received the COVID-19 vaccine during the pandemic, and there was no difference between psychiatric diagnoses (46). In a study conducted in Turkey, the rate of those who received at least 1 dose of vaccine was reported to be 83.7%, and no difference was reported between vaccination status and sociodemographic characteristics such as type of psychiatric diagnosis, age, and education level (24). In our study, it was found that the rate of COVID-19 vaccination during the pandemic was significantly higher in those over the age of 40 and those with a university or higher education level. A correlation was found between psychiatric diagnosis and vaccination status; the highest vaccination rate was found in the patient group diagnosed with ADHD (90.9%), followed by patients diagnosed with bipolar disorder (81.1%) and depression (80.0%); the group that stated that they were vaccinated the least were patients diagnosed with psychosis (54.5%) and OCD (64.3%). In Belgium, COVID-19 vaccination rates among patients with mental disorders were reported to be as high as in the general population (46). In a cohort study examining the electronic health records of 2.8 million people in the UK, COVID-19 vaccination rates of people with psychiatric diagnoses were found to be higher compared to the healthy control group (68.4%); the highest rates were found in people with recurrent major depression (77.1%), followed by those with bipolar disorder (75.7%); it was reported that people with psychotic disorder had the highest vaccine refusal rates (47). In a review examining possible predictors of COVID-19 vaccine hesitancy, schizophrenia was defined as a risk factor (29). Küçükparlak found that having a diagnosis of schizophrenia increased the risk of not getting the COVID-19 vaccine 2.7-fold (30). In a study conducted in Japan, generalized trust, mental health factors such as depression and generalized anxiety, and fear of COVID-19 were identified as predictors for COVID-19 vaccine hesitancy (31).

In this study, according to the scores obtained from the vaccine hesitancy scale, hesitation about vaccines was highest in patients diagnosed with psychosis, depression, and anxiety disorder, respectively, and the lowest level of hesitation was found in patients diagnosed with ADHD and bipolar disorder. The higher vaccine hesitancy in patients with psychosis, depression, and anxiety disorder may be due to the effect of these disorders on the cognitive function, risk perception, and decision-making processes of these individuals. Paranoid ideation or lack of trust in psychotic disorders, low energy levels in depressed individuals, and fears and concerns arising from misinformation about the vaccine in individuals with anxiety disorders may play a role in increased hesitancy. Therefore, specific strategies should be developed for different psychiatric diagnoses when combating vaccine hesitancy in patients with psychiatric disorders. In a study conducted in Turkey, it was observed that unvaccinated patients with schizophrenia stated that they thought that vaccines had serious side effects and caused homosexuality, and it was stated that the vaccination attitude in patients with schizophrenia might be influenced by conspiracy theories (30). In another study conducted in Turkey, a positive correlation was reported between the belief in conspiracy theories and vaccine hesitancy (48). A study evaluating psychiatric morbidity and COVID-19 vaccine hesitancy emphasized that vaccine hesitancy was more prevalent across all psychiatric comorbidities (22). Considering that vaccine hesitancy increases the risk of depression, peritraumatic stress, and anxiety even in a healthy population without a psychiatric diagnosis (49), it should be taken into account that possible increased vaccine hesitancy may increase the clinical burden of disease in patients with psychiatric disorders who may be under high stress and anxiety due to their existing psychiatric diagnoses during periods when the safety of vaccines is discussed more, such as pandemics. In addition, in our study, it was shown that vaccine hesitancy was higher in women and those with less than a university education. Furthermore, there was a positive relationship between vaccination behavior and vaccine hesitancy and vaccine hesitancy was lower in those who received regular influenza vaccination within 1 year and every year, those who received pneumococcal vaccination, and those who received a COVID-19 vaccination during the pandemic. The effect of concern about the safety of COVID-19 vaccines on the intention to be vaccinated has been shown (50). In Lorenz’s study, it was stated that those who perceived a greater degree of vaccine effectiveness were more likely to obtain flu vaccination (19).

Increasing the vaccine uptake of these patients with psychiatric disorders will contribute positively to their life expectancy and quality. Adding preventive care healthcare units to mental health centers where vaccination can also be performed (51) and reminding patients of vaccination by sending messages or emphasizing the importance of vaccination may increase vaccine uptake. There is evidence that integration of mental health and community mental health center services can have a profound positive impact on the severe and persistent mental illness population (52). Including the vaccination status of patients in the patient assessment and follow-up protocols of psychiatrists may increase the awareness of vaccination in patients and contribute positively to vaccination rates and negative vaccine hesitancy. In the Lorenz study, it was found that those who received vaccine education from their doctor were 4 times more likely to be vaccinated for influenza (19).

Limitations of our study include the possibility of recall bias due to the fact that it was a single-center and cross-sectional survey study and that the participant group consisted of a population already suffering from a psychiatric illness and whose recall or ability to comment could be affected due to their illness or medications.

## Conclusion

In conclusion, in our study, we evaluated the vaccination rates and vaccine hesitancy of patients with psychiatric disorders and found that COVID-19 vaccination rates among this group were low and influenza and pneumococcal vaccination rates were extremely low. Our findings clearly demonstrate the need for strategic interventions for the patient group, and the relationships between vaccination status and vaccine hesitancy and sociodemographic characteristics emphasize the importance of planning interventions taking into account psychiatric diagnoses and sociodemographic characteristics of individuals. Although more research has been conducted on COVID-19 vaccination rates of individuals with psychiatric disorders during the pandemic, data on vaccination rates against respiratory infections, especially pneumococcal vaccines, are limited. Therefore, there is a need to support the literature with more studies in this field.

## Data Availability

The raw data supporting the conclusions of this article will be made available by the corresponding author upon reasonable request and following approval by the ethics committee.
